# Adolescents’ perceptions and experiences of pregnancy in refugee and migrant communities on the Thailand-Myanmar border: a qualitative study

**DOI:** 10.1186/s12978-018-0522-7

**Published:** 2018-05-22

**Authors:** Carine Asnong, Gracia Fellmeth, Emma Plugge, Nan San Wai, Mupawjay Pimanpanarak, Moo Kho Paw, Prakaykaew Charunwatthana, François Nosten, Rose McGready

**Affiliations:** 1 Centre for Tropical Medicine and Global Health, Nuffield Department of Medicine, University of Oxford, Oxford, OX3 7FZ UK; 2 Nuffield Department of Population Health, University of Oxford, Old Road Campus, Headington, Oxford, OX3 7FZ UK; 3 Shoklo Malaria Research Unit, Mahidol-Oxford Tropical Medicine Research Unit, Mahidol University, Mae Sot, 63110 Thailand; 4 Mahidol-Oxford Tropical Medicine Research Unit, Mahidol University, Bangkok, 10400 Thailand

**Keywords:** Adolescent pregnancy, Refugee, Migrant, Myanmar, Qualitative, Sexual and reproductive health, Contraception, Stigma, Forced marriage, Domestic violence

## Abstract

**Background:**

Adolescent pregnancy remains a global health concern, contributing to 11% of all births worldwide and 23% of the overall burden of disease in girls aged 15–19 years. Premature motherhood can create a negative cycle of adverse health, economic and social outcomes for young women, their babies and families. Refugee and migrant adolescent girls might be particularly at risk due to poverty, poor education and health infrastructure, early marriage, limited access to contraception and traditional beliefs. This study aims to explore adolescents’ perceptions and experiences of pregnancy in refugee and migrant communities on the Thailand-Myanmar border.

**Methods:**

In June 2016 qualitative data were collected in one refugee camp and one migrant clinic along the Thailand-Myanmar border by conducting 20 individual interviews with pregnant refugee and migrant adolescents and 4 focus group discussions with husbands, adolescent boys and non-pregnant girls and antenatal clinic staff. Inductive thematic analysis was used to identify codes and themes emerging from the data.

**Results:**

Study participants perceived adolescent pregnancy as a premature life event that could jeopardise their future. Important themes were premarital sex, forced marriage, lack of contraception, school dropout, fear of childbirth, financial insecurity, support structures and domestic violence. Supportive relationships with mothers, husbands and friends could turn this largely negative experience into a more positive one. The main underlying reasons for adolescent pregnancy were associated with traditional views and stigma on sexual and reproductive health issues, resulting in a knowledge gap on contraception and life skills necessary to negotiate sexual and reproductive choices, in particular for unmarried adolescents.

**Conclusions:**

Adolescents perceive pregnancy as a challenging life event that can be addressed by developing comprehensive adolescent-friendly sexual and reproductive health services and education in refugee and migrant communities on the Thailand-Myanmar border. Creating a more tolerant and less stigmatising environment in these communities and their governing bodies will help to achieve this goal.

## Plain English summary

Globally 17 million girls under the age of 19 give birth every year, among them one million under the age of 15. Early pregnancy and motherhood put these adolescents at a higher risk of death, health problems, social stigma, school dropout, unemployment, poverty and domestic violence. Their babies are more likely to die and to have long-term health problems.

This study was set up to get a better understanding of the views and experiences of adolescent pregnancy in refugee and migrant communities on the Thailand-Myanmar border, where poverty, poor education and health facilities, early marriage, difficult access to contraception and traditional beliefs may cause particular difficulties.

The investigators conducted 20 interviews with pregnant refugee and migrant adolescents, as well as four focus group discussions with husbands, adolescent boys and non-pregnant girls and antenatal clinic staff.

Most of the adolescents agreed that unplanned teenage pregnancy is a negative life event during a time that needs to be spent in school. Important themes were premarital sex, forced marriage, lack of contraception, school dropout, fear of childbirth, financial insecurity, insufficient support and domestic violence. Supportive relationships with mothers, husbands and friends were considered to be helpful.

Among the reasons for adolescent pregnancy were traditional views and stigma on sexual and reproductive health issues, resulting in a knowledge gap on contraception and life skills necessary to negotiate sexual and reproductive choices, in particular for unmarried adolescents. Better adolescent-friendly sexual and reproductive health services and education can address this gap in refugee and migrant communities on the Thailand-Myanmar border. In addition a more tolerant and less stigmatising environment could encourage open communication between adolescents and their families and within sexual relationships.

## Background

Adolescent pregnancy is an important global health issue and is linked to seven of the Sustainable Development Goals (SDG 1–5, 8, 10) [1]. Every year an estimated 16 million girls aged 15–19 years and another one million girls under the age of 15 give birth; 95% of these births occur in low- and middle-income countries, with the highest rates in sub-Saharan Africa and south Asia. Births to adolescents under 20 years represent 11% of all births worldwide but 23% of the overall burden of disease in this age group [2].

Complications of pregnancy and childbirth, including unsafe abortions, make pregnancy one of the leading causes of death in adolescent girls. Health problems such as anaemia, malaria, sexually transmitted infections, in particular HIV, postpartum haemorrhage and mental disorders are strongly associated with negative outcomes of adolescent pregnancy. About 65% of all obstetric fistulae develop during adolescence, generating significant physical, social and psychological consequences. In addition infants of adolescent mothers are more likely to die, have low birth weight and experience long-term adverse health effects in comparison with infants of adult mothers [2–4]. Premature motherhood significantly reduces the chances of continuing education, developing skills and finding paid work, thus creating a negative cycle of adverse health, economic and social outcomes [5]. A large body of evidence has highlighted the damaging consequences for adolescent mothers and their babies [6–9].

Although some adolescents want and plan their pregnancy, for most of them pregnancy is unplanned and associated with poverty, living in rural areas, difficult access to health care and family planning, early marriage and lack of (sexual and reproductive health) education [3]. Migrant and refugee adolescent girls may be even more at risk [10, 11]. Numerous married adolescent girls cannot access existing family planning services because their husbands or family do not approve due to traditional beliefs. Unmarried adolescents may have particular difficulties accessing contraception because premarital sex is judged as inappropriate and unacceptable [12–14].

**Fig. 1:**
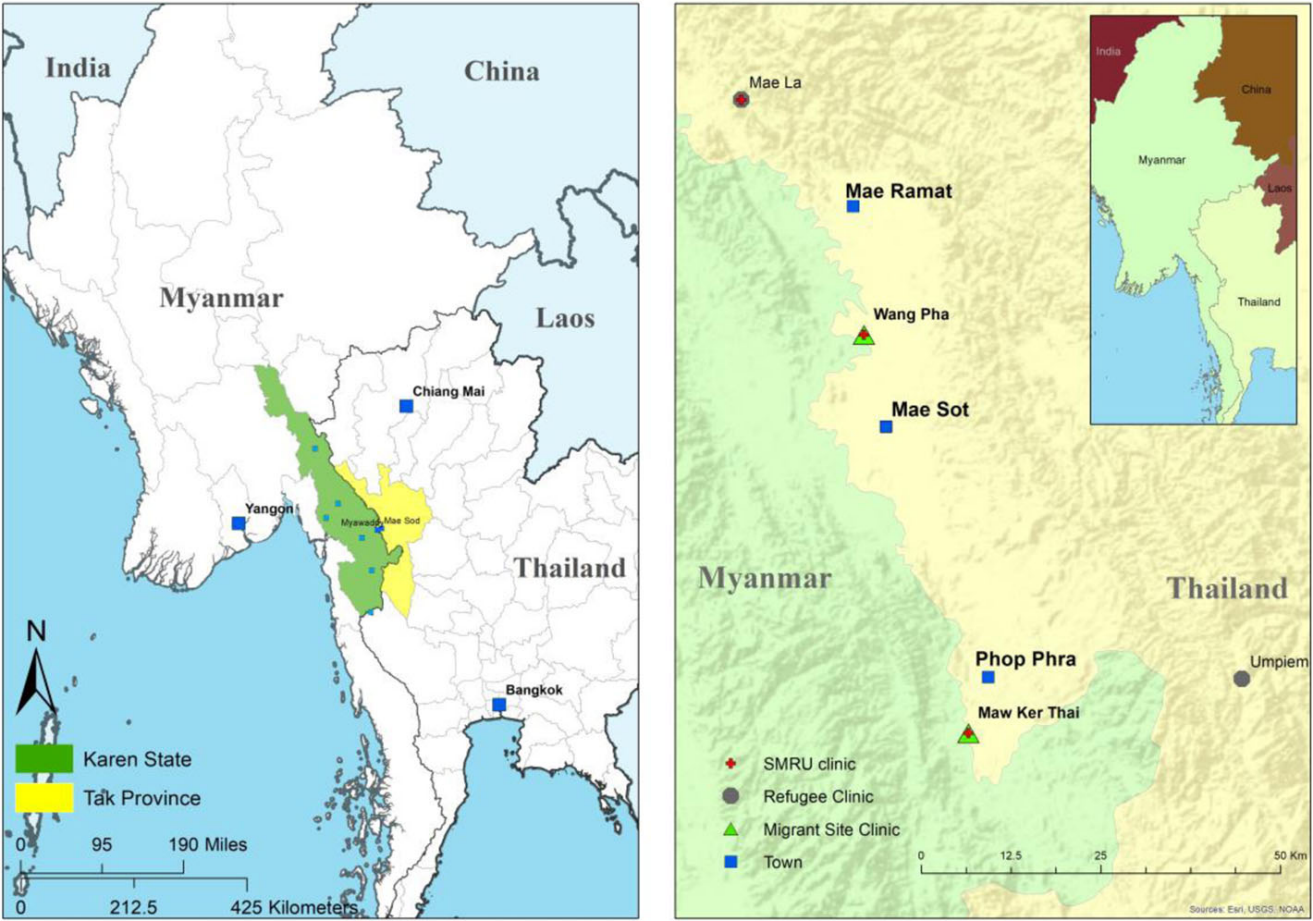
Map of study area (Credit to Myo Chit Min, Shoklo Malaria Research Unit)

The Thailand-Myanmar border (Fig. 1) is home to large populations of refugees who initially fled civil war in Myanmar [15]. Currently an estimated 100,000 refugees live in ten camps on the Thai side of the border. For the past 10 years camp life has been relatively safe and stable, but education, employment and access to the outside world are restricted [16]. Despite poverty and confinement, people feel safe and are reluctant to leave, now that voluntary return and relocation programmes have been negotiated between the Thai and Myanmar governments, envisioning closure of all refugee camps within a not yet defined time period [17].

This region also has an estimated 200,000 migrant workers from Myanmar who have come to Thailand for employment in agriculture or construction, earning higher salaries than in Myanmar [18, 19]. Migrants remain at risk of arrest, extortion and deportation unless they or their employer can afford official registration. The migrant and refugee populations in this region include many ethnic groups such as Karen, Burman and Burman Muslim, each with their own culture, language and religious backgrounds. In this area a number of non-governmental organisations (NGOs) provide basic humanitarian, education and health services, including prenatal care [16].

Little is known about adolescent pregnancy and motherhood in this setting. Previous research has focused on pregnant adolescents with refugee backgrounds after resettlement in developed countries [20, 21]. A 2010 study in two refugee camps on the Thailand-Myanmar border assessed young refugees’ reproductive health knowledge, attitudes and sources of information and identified early marriage as a major problem [22]. The aim of the current study was to develop a better understanding of adolescent pregnancy, including sexual and reproductive health knowledge and family and community support structures on the Thailand-Myanmar border. In this paper we report on the findings relating to the adolescents’ perceptions and beliefs of early motherhood.

## Methods

### Setting

Since 1986 the Shoklo Malaria Research Unit (SMRU) has provided free antenatal care to displaced populations on the Thailand-Myanmar border and has extensive experience of working with local refugee and migrant communities. The antenatal clinic (ANC) in Mae La refugee camp (MLA) serves the refugee community in the largest camp on the Thai side of the border, while the Maw Ker Thai (MKT) ANC serves rural migrant communities on both sides of the border, south of Mae Sot, Thailand. ANC staff consist mainly of locally trained medics, midwives, nurses and counsellors, supported by a minimal international team of clinicians and researchers [23]. Based on clinic data an estimated 16–18% of all pregnant women seen at SMRU ANC are adolescents under the age of 18.

### Study design [24]

Pregnant adolescents younger than 18 years were recruited for in-depth interviews. Eligible participants were pregnant adolescents who registered for a first antenatal visit between 1 September 2015 and 15 June 2016 in the MLA or MKT ANC. A total of 76 adolescents were identified in the clinic database, 40 in the MLA ANC and 36 in the MKT ANC, using convenience sampling, i.e. we selected participants who were coming to the clinic for antenatal visits during the data collection period. A senior midwife approached them individually in the ANC waiting area to explain the purpose of the study and explore their interest in participating.

Additional focus group discussions (FGDs) were held in MLA with husbands of pregnant adolescents, adolescent boys and non-pregnant girls younger than 18 years, and locally trained ANC staff members. Participants for the FGDs were recruited by various methods. Husbands of pregnant adolescents were recruited through snowballing, by asking pregnant adolescents to invite their husbands to attend the clinic on a Saturday morning. Adolescent boys were recruited in a MLA boarding school, where students from villages outside the camp on both sides of the border receive free education, while non-pregnant adolescent girls were recruited by asking locally trained ANC staff members to invite their adolescent daughters and friends. ANC staff members rostered onto the daytime shift were recruited on a voluntary basis after a professional skills training for ANC staff of different camps.

All sessions were conducted in private meeting rooms in proximity to the ANC waiting area and planned to last between 45 and 60 min. Since four different languages are spoken in this setting (Sgaw Karen, Poe Karen, Burmese and English), all interviews and FGDs were co-facilitated by the principal investigator (CA) and a senior midwife (NSW in MLA, MP and MKP in MKT). Topic guides were discussed with facilitators beforehand to check cultural appropriateness and familiarise them with the content. Similar topic guides were used for the interviews and FGDs with husbands and adolescent boys and non-pregnant girls. For the FGD with ANC staff some extra questions were added to explore their willingness to discuss contraception with (unmarried) adolescents (Table 1). The facilitators translated the questions into Karen or Burmese and the participants’ responses into English, while the principal investigator took notes to enhance later data analysis and interpretation. All interviews and FGDs were audio recorded for future reference. Participants received a towel and a bar of soap at the end of the interview or FGD as a token of thanks.

**Table 1:** Interview/FGD topic guide

1. At what age most women become pregnant? Do you think this is young/old to have a baby? What is a young age to have a baby? What advice do you give to your best friend on the best age for pregnancy? 2. Why do women become pregnant at a young age? Are the fathers also young/older? Who decides to have a baby? Father? Mother? Together? 3. Which things can make pregnancy and motherhood difficult? Which things can make pregnancy and motherhood easier? Can you give examples? Can you explain more? 4. Is pregnancy/motherhood at a young age different from pregnancy/motherhood at an older age? How is it different? Can you give examples? 5. Do you know pregnant adolescents or young mothers who are not happy and lonely? Why do you think they are not happy and lonely? How do you know they are not happy and lonely? 6. Who can help these young pregnant women/mothers when it gets difficult? Could you explain how that will help young pregnant women/mothers? What can happen if they have nobody to help them? 7. Have you heard of ways to prevent or stop pregnancy? Can you give examples? If adolescents can get contraception, will they use it? Can you explain why they will use it/ not use it? 8. What do schools teach young girls about pregnancy, contraception? Do you want schools to teach young girls about pregnancy, contraception? Where can they find this information? Can you explain that more? Can you give examples? 9. What do young men know about pregnancy/contraception? Do they need more information/education on pregnancy/contraception? Why do you think that is important? Where can they find this information? 10. FGD ANC staff only: Can you describe ways to improve the knowledge of adolescents about sexual health, pregnancy and contraception? Are you comfortable discussing this with adolescents? Would you like to learn more about this?	

Notes from the interviews and FGDs were reviewed and discussed with the facilitators at the end of each day of data collection. Due to time limitations data saturation was not formally aimed for and assessed.

A Karen refugee student (DK) transcribed all voice recordings and translated them into English. Complete transcripts of the interviews and FGDs were only available at the end of the data collection period. A senior administrative SMRU staff member performed accuracy checks and back-translations of a random 10% of transcripts. NVivo for Mac v11.3.1 was used to organise the data set and prepare it for thematic analysis. Inductive thematic analysis was conducted in six consecutive steps, as outlined by Braun and Clarke [25, 26]. Two authors (CA and EP) separately coded the transcripts and agreed on key emerging themes.

### Ethics

Participant information and informed consent/assent were administered verbally to all participants. Adolescents under the age of 18, the legal age of consent in Thailand, were invited to provide written assent, co-signed by a parent, husband, sister or guardian prior to participation in the study. Low-literacy participants provided a thumbprint instead of a signature. A copy of the consent/assent form was given to each participant. Ethical approval for this study was obtained from the University of Oxford’s Tropical Research Ethics Committee (OxTREC Ref [13–16]), the Mahidol University Faculty of Tropical Medicine Ethics Committee (TMEC16–025) and the Tak Border Community Advisory Board (T-CAB-04/01/2016).

## Results

We conducted a total of 20 individual interviews with adolescents who were pregnant at the time of recruitment: ten in the MLA and ten in the MKT ANC. In addition four FGDs were conducted in MLA only: FGD1 with three husbands of pregnant adolescents, FGD2 with six adolescent boys, FGD3 with six non-pregnant adolescent girls and FGD4 with five locally-trained ANC staff members. All eligible adolescents and staff members who were approached for an interview or FGD agreed to participate and signed a consent form. No participants left during any of the interviews, but one participant of FGD1 left after 5 minutes without providing a reason. The duration of the interviews varied between 14 and 45 min. The FGDs were longer and lasted between 62 and 115 min. Tables 2 and 3 give an overview of the socio-demographic characteristics of the study participants.

**Table 2:** Socio-demographic characteristics of interview participants

Reference number^a^	Age of participant	Age of husband	EGA (weeks)^b^	Language	Years in school	Prior employment
MLA01	16	18	8	Burmese	4	No
MLA02	15	15	38	Burmese	2	Shop auntie
MLA03	16	25	19	Karen	11	Agricultural work
MLA04	15	20	12	Burmese	4	Home shop
MLA05	16	25	Post-partum	Karen	5	Household
MLA06	15	18	Miscarriage	Karen	3	Agricultural work
MLA07	15	19	27	Karen	4	Care siblings
MLA08	17	18	31	Karen	9	Agricultural work
MLA09	17	25	34	Burmese	3	Agricultural work
MLA10	14	15	–	Karen	8	No
MKT01	16	18	8	Burmese	4	Agricultural work
MKT02	15	25	26	Burmese	0	Agricultural work
MKT03	17	20	36	Burmese	2	Agricultural work
MKT04	16	23	18	Karen	3	Agricultural work
MKT05	13	20	36	Karen	0	Agricultural work
MKT06	17	22	17	Burmese	4	Agricultural work
MKT07	16	20	22	Burmese	9	Agricultural work
MKT08	16	19	16	Karen	4	Agricultural work
MKT09	15	18	25	Karen	6	Agricultural work
MKT10	17	24	20	Karen	7	Household

^a^MLA: participants attending antenatal clinic in Mae La refugee camp; MKT: participants attending antenatal clinic in Maw Ker Thai migrant clinic

^b^EGA estimated gestational age at time of interview

**Table 3:** Socio-demographic characteristics of FGD participants

Reference number	Age of participant	Years in school	Employment	Language
Husbands of pregnant adolescents				
1FGD1	16	3	Agricultural work	Karen
2FGD1	15	7	No	Karen
3FGD1	18	11	Teacher	Karen
Adolescent boys				
1FGD2	14	7	Student	Karen
2FGD2	19	10	Student	Karen
3FGD2	17	3	Student	Karen
4FGD2	16	9	Student	Karen
5FGD2	16	8	Student	Karen
6FGD2	18	5	Student	Karen
Adolescent non-pregnant girls				
1FGD3	14	7	Student	Karen
2FGD3	17	10	Student	Karen
3FGD3	16	9	Student	Karen
4FGD3	13	6	Student	Karen
5FGD3	16	9	Student	Karen
6FGD3	16	9	Student	Burmese
Antenatal staff (all locally trained)				
1FGD4	–	–	Ultrasound technician	Karen
2FGD4	–	–	Midwife	Karen
3FGD4	–	–	Nutrition counsellor	Karen
4FGD4	–	–	Midwife	Karen
5FGD4	–	–	Midwife	Karen

A preliminary analysis of the data transcripts showed that similar themes were emerging from the individual interviews and FGDs, as were refugee and migrant participants’ views, and therefore all results were analysed together.

Two overarching themes emerged, “getting pregnant” and “becoming a mother”, each including different levels of closely interrelated sub-themes.

### Getting pregnant

#### Too young

All adolescents agreed that they were too young for pregnancy and motherhood and that a minimum age of 20 was more appropriate. While adolescent girls worried mostly about physical difficulties in childbirth and financial hardship, adolescent boys thought that missed educational opportunities and social consequences could make the future difficult. One participant in the husbands’ focus group noted that it was problematic for young fathers under the age of 20 because ‘their education is still too low and they are not ready to take responsibility for everything.’

*“If girls get pregnant and become mothers at such young age, it is difficult for them, like … not having enough money… If a husband cannot earn some money, it will be a problem.” (MLA10 pregnant refugee)*

#### Marriage

In this context premarital sex and pregnancy outside of marriage are disapproved and not tolerated by general society and culture. As is common in many rural and traditional societies, interaction between unmarried boys and girls can only take place in public or in the presence of a family member or friend, in particular in the evening hours, when adolescents are expected to experiment with sexuality. Consequently a rendezvous between two young people without a chaperone can result in a forced cover-up marriage, sometimes even within the next 24 h. ANC staff members clearly described the stigma that is associated with premarital sex in their community.

*“So how does the community react when they catch young couples sleeping together, they force them to get married, even if they don’t agree. Once they’re married, then the issue is over… the people don’t talk about it anymore. You will be dead if you don’t get married after you’ve slept together!” (FGD4 ANC staff)*

Talking about premarital sex was an extremely sensitive topic for most adolescents. They avoided the words, indicated that they did not wish to discuss it, replied by ‘I don’t know’ or referred to it in different ways. Out of the 20 interviewed pregnant adolescents, four refugees and three migrants indicated that they were forced to marry after they got pregnant. As one pregnant refugee noted, ‘Some end up getting pregnant because they indulge themselves in the wrong track in their lives.’

However some pregnant refugees and migrants explained that they themselves decided to marry and that free-choice adolescent marriages out of love were more common than arranged and cover-up marriages. A 15-year old pregnant refugee, who lived most of her life in Myanmar before moving to Mae La camp, defended a rather liberal viewpoint, similar to that of most western countries and very unusual in this conservative setting, stating that ‘couples just decide to live together and get married when the girls become pregnant.’

For one refugee girl marriage was a coping strategy to escape a poor and abusive home environment. She ran away in search for a better life in the Bangkok area, where she married a co-worker. The unplanned pregnancy however forced her to return to Mae La and live with her parents again.

*“After my father left, my mother married another man. He does not like me and he always yelled at me. I had to do all the hard work in the house, so I ran away.”*

*“I didn’t plan for this, my pregnancy, but it just happened. And my husband told me that we would not get pregnant until we were financially doing well. If we have the baby now, both for us and for the baby, we’ll be in trouble. My mother has to live with her husband and if she wants to help me or give me money she needs to get permission from my stepfather. When I feel sad, I have to make myself feel better, because I cannot talk to my mother anymore.” (MLA09 pregnant refugee)*

Marriage at a young age, arranged by parents or other family members, is less common in this setting and occurs mostly in Muslim families. One interviewee explained that her widowed mother had married her off at 13 years of age to secure support for her family.

#### Planned or unplanned pregnancy?

Of all interviewees only two migrant girls (16 and 17 years old) confirmed that they made a positive choice to marry and have a baby after prior use of contraceptives. More than half of all pregnancies took the young couples by surprise. It ‘just happened’, mostly because they were not using contraception or their contraception failed due to irregular use.

*“My husband’s aunt saw me and asked me how long I had missed my period? I told her about two months. So she asked me if I were pregnant and I told her that I didn’t know.” (MLA09 pregnant refugee)*

In only one case, an arranged Muslim marriage, a religious explanation was given for the unplanned pregnancy.

*“It is just the way it is because God gives blessings to them for their pregnancies.” (MLA01 pregnant Muslim refugee)*

Some adolescent girls had to give in to the strong wish of their husband or parents to have a (grand) child.

“*After the marriage I took some pills. But my husband told me to stop … his mother wanted him to have a baby. Then the whole family discussed together and made the final decision.” (MKT08 pregnant migrant)*

*“However now I have to follow the decisions by my husband because I’m very young. You can’t say ‘no.’ … It’s just …” (MKT09 pregnant 15 year-old migrant)*

There was however a fine line between forced sex and a husband’s wish to have a baby. Some girls indicated that they were not assertive enough to negotiate reproductive choices with their husbands. This was too sensitive an issue to explore further in the individual interviews, but it was discussed in the adolescent boys’ FGD2.

*Q: “For example, a boy asks his girlfriend to sleep with him but the girl refuses, what do you think of this situation?”*

*A: “It would be forced sex and the boy should respect the girl’s decision. A boy shouldn’t force her to sleep with him because it would be a kind of rape, although they are boyfriend and girlfriend. It’s so wrong!” (FGD2 adolescent boys)*

#### Contraception

The knowledge and beliefs about contraception play a crucial role in adolescent pregnancy in this setting and are tightly interwoven with the taboo around sexual and reproductive health (SRH) education for unmarried adolescents.

*“So it is difficult to talk to unmarried boys and unmarried girls about family planning and safe sex. People do not accept it because they think that such education can corrupt their children’s minds, like they think it teaches them to be more sexually active. We used to provide family planning education to the community. When we showed for example, how to use condoms, some girls felt embarrassed about it, even the married people … they never looked at that demonstration! If even the married people felt very embarrassed, you can imagine the unmarried people.” (FGD4 ANC staff)*

SRH education in camp and village schools is limited to biological facts on puberty and pregnancy and does not include any information on contraception, sexual relationships and life skills.

*“Sometimes teachers teach us about reproductive stages, a female’s thing and a male’s thing. We don’t get to learn about sex and pregnancy or family planning. We only know that from neighbours.” (FGD3 adolescent girls)*

*“In school I only learned about menstruation and the boys laughed about this when the girls were explained …” (MLA02 pregnant refugee)*

*“We don’t know how to make the right decisions and we do not talk about it with our wife. But school is not a good place to teach about contraception. I think it can give the students bad habits, if they know about prevention.” (FGD1 husbands)*

Most adolescents received information on contraception elsewhere, from mothers, sisters, aunties, neighbours and friends. Some adolescents preferred to talk to friends only, because they dreaded their parents’ judgement when showing an interest in contraception. One participant in the adolescent girls’ focus group was clear that ‘we don’t feel comfortable to talk about this with our mothers: without getting married, talking and discussing with our mothers about this is a bit … our mothers may think that their daughters are pregnant … we will end up being scolded.’ Only a few participants mentioned health professionals as the best resource.

Many adolescents had heard about contraceptives, but did not know how to use them correctly. A common misconception was the idea that the use of contraception before a first pregnancy causes the uterus to shrink.

*“Umm… I have heard that taking contraception pills can sometimes lead to uterus contraction, and a girl can no longer conceive a baby after that.” (MLA04 pregnant refugee)*

Most antenatal and family planning clinics offer information on contraception and provide condoms, contraceptive pills and injectable hormones free of charge. However sometimes staff members refuse to provide contraception to unmarried adolescents.

*“They seem to only give medications and condoms to the married couples. If some of them are still in school, they wouldn’t be given anything to prevent pregnancy.” (MLA08 pregnant refugee)*

Adolescents are hesitant to use the clinic services because of the associated stigma and social control vis-à-vis premarital sex: a clinic visit in itself could show the community that the adolescent is sexually active or interested in premarital sex. A participant in the adolescent boys’ group stated that, ‘I feel shy for getting a condom, we feel a bit uncomfortable to get condoms from the health-workers working in our villages.’

Often adolescents ask a relative or friend to get contraceptive pills or condoms on their behalf in pharmacies and regular shops because they do not want to be caught in the act of buying contraceptives.

*“For contraception it is better to live in a big city. Nobody will know you there when you want to buy contraceptives or condoms. In the village everybody will know and they will talk.” (FGD2 adolescent boys)*

#### School dropout

Across all interviews and FGDs adolescent pregnancy was believed to cause major disruptions in the lives of unmarried adolescents. Some schools expelled students if they knew about premarital sex while other schools allowed students to stay if they got married. However the pregnant adolescents and their partner always chose to leave school, some because they felt embarrassed and teased by their classmates, others because the new responsibilities as parents caused pressure to find work and support the young family.

*“I didn’t want to go to school anymore because I feel embarrassed about me getting married earlier. Some of my friends seem a bit different, looking down at me like ‘oh, you’re a married man now…’ They tease me like that. And it is the same for the girls.” (FGD1 husband)*

*“ In my point of view, once you get married, you have a different life. So for me, I have to work for my life and family now when my friends are going into different directions of life. Now, I have to take responsibility for my family.” (FGD1 husband)*

While the boys voiced more feelings of regret about missed educational opportunities, the pregnant girls seemed to have less of a problem with this school dropout and some really cherished the thoughts of motherhood,.

*“Since I’m married, I’ll need to take care of the baby soon and I don’t want to be in school anymore.” (MLA08 pregnant refugee)*

### Becoming a mother

Most interviews and FGDs were saturated with negative experiences, stories and examples. Few participants raised positive experiences spontaneously and most of them only talked about them when asked for explicitly.

#### Wellbeing of mother and baby

For most pregnant adolescents feeling healthy and physically comfortable seemed to give them more confidence and resilience towards the future. Knowing that the baby was healthy and growing well made them happy, especially when sharing and comparing their bellies with some of the other pregnant women in the ANC waiting room.

*“I have two new friends in my neighbourhood and they are also pregnant. We come to the clinic together; there is one in the waiting room over there. We often talk about our babies and how they get bigger.” (MLA08 pregnant migrant)*

Besides nausea and minor pregnancy-related ailments there were few examples of physical difficulties during adolescent pregnancy. The only important topic that emerged from the interviews and discussions was physical labour. Most pregnant migrants had been working in the fields and indicated that they had already stopped or were planning to stop their work soon. One pregnant refugee however complained that she had to work hard in the third trimester of her pregnancy to ‘pay’ for support from her relatives. To protect her in the last weeks before childbirth she was admitted to the antenatal ward by the local midwives.

*“Some women can live comfortably during their pregnancy whereas I have to work without any rest at my aunts’ house, including cooking, going out to sell stuff… the whole day. My aunts… they usually come back around 11 o’clock then go out elsewhere … I am left behind to do chores if I want to live in their house.” (MLA02 pregnant refugee)*

#### Necessary support

Perceptions of health and physical comfort were closely linked to the daily living conditions of the young mother-to-be. Having financial support and family around made the pregnancy a lot easier, as one pregnant refugee noted, ‘it is all easy for me, because my uncles and aunts from abroad support me. And I also have my mother and a younger sister in the house.’

Supportive and caring relationships were seen as indispensable assets. Most pregnant adolescents declared that having family around, especially their mother, was the best support they could wish for. They preferred their mother or female relatives over their husband for emotional and practical support and advice during and after the pregnancy. For more ‘planned’ adolescent pregnancies, based on mature relationships, the support preferences were similar. Mothers were not only perceived as experts in pregnancy and childcare, both ‘women’s businesses’, but even more as unconditional supporters, in spite of social pressure and community disapproval. When mothers were unavailable older sisters and aunties tended to take over this role. On the other hand a good and caring relationship with their husband was perceived as a condition for a stable and financially sound future life. Sometimes the embarrassment of adolescents towards their family was just too overpowering and friends were the first resource for emotional support.

*“Talking to mother is still the best thing to do because yes, she may scold you, but she will finally take care of you.” (FGD3 adolescent girls)*

The young fathers perceived it as a duty to work and protect their young wives. They acknowledged the importance of family support for the young mothers, but some also emphasised the adolescent father’s need for knowledge and involvement in baby care.

*“But young fathers should also receive support about caring for their baby if they want to be involved. But in my opinion, the young father needs to support his wife in physical things… She always needs company with her when going somewhere at night too, for safety, I mean.” (FGD1 husbands)*

#### Perceived challenges

Nearly every pregnant adolescent expressed fear of physical difficulties and pain during childbirth. They had all heard that having a baby at a young age can be difficult and that a Caesarean section is sometimes necessary. The financial burden of an operation in the Mae Sot hospital represented an additional concern for these young women and their families.

One migrant’s story was very emotional because she was only 13 years old.

*“When I first got pregnant, I thought of myself that I was going to die because I’m young. I was worried and cried. Then I talked to my mother. My mother just told me ‘it’s nothing’. And my husband said that it is a girl’s thing and that I have to be ready for the pain.” (MKT05 pregnant migrant)*

Many of the pregnant adolescents worried about financial hardship in the immediate and far future. Most pregnant refugees had been in school until they got pregnant. Without financial support from their parents, relatives or their husbands, they would have to find work to support their family, a difficult task for a woman living in Mae La refugee camp.

*“I didn’t have any money for today to cook, I didn’t have rice. Then I had to go ask some from others and if they didn’t give me some rice, I had to go to some other places to find food.” (MLA09 pregnant refugee)*

Apart from two pregnant migrants who planned their pregnancies, feelings of unpreparedness, struggle and unhappiness emanated from many stories. Instead of completing their education in school and spending time with friends, they had to dedicate their life to a baby, organise their household or earn a living. In some stories young mothers refused to accept this new role, disliked and even neglected their baby.

*“I have seen a very young pregnant girl, about 14 years old, around my house. She gave birth to a big and healthy baby at the hospital. However, about two weeks after she was discharged from the hospital, she went out to join her friends and play. When she came back her baby was already dead. Some of her neighbours felt sorry for what just happened, but she kind of felt nothing.” (MLA08 pregnant refugee)*

Some pregnant adolescents admitted that they had considered abortion as a solution to their unplanned pregnancy, but fear and traditional beliefs prevented them from carrying it out.

*“In my village, we believe that when a person tries to make an abortion and has succeeded, afterwards in her next life, she has to eat back the things she aborted in a previous life. Also you cannot get pregnant anymore! That’s why it scared me!” (MKT09 pregnant migrant)*

#### Domestic violence and suicide

Unplanned adolescent pregnancy and the subsequent cover-up marriage were generally perceived as major risk factors for troubled relationships both within families and within marriages. Social pressure and traditional culture kept some parents from supporting their pregnant daughter and made them use violence instead to solve the problem. One adolescent boy related a sad story about one of his friends who ‘was beaten by her mother because she didn’t like her daughter getting pregnant early, so she lost the baby.’

Numerous were the stories about husbands not supporting their young pregnant wife or family, often resorting to alcohol and drug abuse, gambling, domestic violence and divorce.

*“She loves her husband, but her husband doesn’t love her. She lives with her mother-in-law, but that mother-in-law treats her badly. Her mother-in-law and her husband always beat her up. Her husband drinks alcohol, uses drugs… umm… he treats his wife badly.” (MKT03 pregnant migrant)*

In more extreme situations suicide was perceived as the only way out.

*“For other people, their husbands are not good to them. Sometimes they commit suicide as they feel young and are in such a complicated situation. Their husbands drink alcohol a lot and there is domestic violence, so that the wives do something when becoming depressed … like committing suicide by drinking poison.” (MLA02 pregnant refugee)*

## Discussion

In the refugee and migrant communities on the Thailand-Myanmar border adolescents perceive pregnancy as a premature and usually unplanned life event that can jeopardise their future chances in life. Pregnant adolescents are fearful of childbirth and premature motherhood, but most of all the uncertainty to secure a well supported and financially sound future. These findings endorse the large body of evidence that highlights the disadvantages and risks of teenage pregnancy and keeps it high on the global health agenda [6–9].

Despite their initial negative reactions, mothers or other women in the family are perceived as a major asset to encourage adolescents during pregnancy and teach them how to be a good mother after childbirth. In this setting many husbands work outside the refugee camp or in the Bangkok area to earn higher wages, and they are physically not present to support their pregnant wives. This may have played an important role in the experiences of marital support that is mostly perceived as a means of financial security. A qualitative study exploring the experiences of teen pregnancy of African Australian refugee women after resettlement in Australia by Ngum Chi Watts et al. [21] describes similar findings. The importance of support structures in teenage pregnancy and parenthood are also highlighted by Letourneau et al. [27] on family and partner support, and Bunting et al. on the role of young fathers [28].

On the other hand, many participants’ stories demonstrate how the lack of support and troubled relationships, including social stigma, domestic violence and alcohol abuse, can drive young families into dire living conditions and young mothers into despair and even suicide. This reflects the findings reported by Fellmeth et al. [29] in the same setting, that economic and family-related causes are the most commonly raised issues among the causes of mental illness. A study by Falb et al. [30] documenting violence against Karen refugee women along the Thailand-Myanmar border, reports that 7.9% of all surveyed women, irrespective of age, were recent victims of intimate partner violence often linked to alcohol abuse. Fear of women to speak out could have caused underreporting and the real numbers were likely to be higher [30]. In our study both refugee and migrant participants reported domestic violence and hence this phenomenon does not seem to be limited to the refugee camp context only.

The main underlying causes for adolescent pregnancies in refugee and migrant communities on the Thailand-Myanmar border can be linked to traditional and stigmatising views on premarital sex. Communication about sexual and reproductive health between parents and unmarried adolescents is mostly perceived as non-existent because it is deemed inappropriate and corrupting young people’s minds, as previously described by Srikanthan et al. [31] in an overview of religious and cultural influences on contraception. Even health workers are reported to refuse contraceptive information and services to unmarried adolescents, similar to the results of Brand et al. [32]. Likewise schools offer a very limited sexual and reproductive health programme, avoiding the topics of contraception, sexual behaviour and life skills necessary to negotiate informed and healthy reproductive choices.

The subsequent adolescent SRH knowledge gap contributes to a highly unmet need for contraceptive services by unmarried adolescents, enhanced by perceptions of social control by the community, in particular in the more isolated refugee camp setting. This corresponds with the findings of Benner et al. [22], demonstrating how youth in refugee camps on the Thailand-Myanmar border are not supposed to have premarital sex or to need reproductive health services, and with a review of contraception barriers for adolescents in low- and middle-income countries by Chandra-Mouli et al. [13]. Once married, adolescents can get information about contraception from their mothers, older siblings or friends, but open communication between husband and wife on reproductive choices is often experienced as uncomfortable and not done. Family planning counsellors and midwives in the antenatal clinics confirm these experiences. In addition they explain how they have to counter deeply ingrained misconceptions and fears for compromised future fertility when they try to recommend contraception for adolescent girls.

### Strengths and limitations

This study is based on limited numbers of individual interviews and focus group discussions, both qualitative research methods that are well suited to explore sensitive and complex social issues. Its findings are not representative of all pregnant refugee and migrant adolescents in this or other settings. Due to time and budget restrictions the study design and recruitment strategy were determined by pragmatic considerations rather than by achieving data saturation.

There was a striking difference in depth, richness and length between the individual interviews and the FGDs, suggesting that the FGD group dynamics were more effective in eliciting stories, perceptions and explicit views on the sensitive topic of teenage pregnancy than individual in-depth interviews. This may be because of the sense of community so that it was more ‘natural’ to speak in groups than to spend a long period of time talking to one person individually. Previous studies confirm that the FGD format works well in the SMRU setting [29] and that Karen refugees in the USA have been notably shy in individual in-depth interviews [33].

All adolescent FGD participants, boys and girls, were secondary school students, who were highly talented and used to voicing their opinion. The unheard voices in this study were the low-literacy, less educated migrant adolescents living in villages and shelters along the border and working in the fields for a living. Future research on this topic should target these groups specifically.

Conducting qualitative research in a setting with different languages may have influenced participants’ responses. The indirectness of communication between an English-speaking researcher and study participants leaves, to a large extent, the contents and flow of conversations into the hands of the facilitators and out of control of the researcher. Three different senior midwives facilitated the interviews and FGDs, each approaching and encouraging the participants in their own style. Notes from the interviews and FGDs were reviewed and discussed with the facilitators at the end of each day of data collection, but we did not perform any cross-checking for accuracy of interpretation of results among the facilitators involved in data collection. A young refugee student translated all participant documents and transcribed all voice recordings with only partial accuracy checks. Due to budget restrictions the transcription of all Karen and Burmese interview and FGD recordings was done directly in English instead of by a two-step process.

Finally, despite rigorous informed consent procedures, adolescent participants may have felt intimidated or afraid to decline participation, particularly in the presence of a non-local researcher.

As far as we can ascertain this appears to be the first publication where the views of pregnant adolescents in Asian refugee camps and amongst Myanmar migrants in Thailand have been directly obtained. The findings of this study can help to identify and support initiatives in the refugee and migrant communities, schools and local health centres to improve adolescent health and empower adolescents towards a healthier life-course, in preparation for the uncertainties of a future return and resettlement to Myanmar or third countries [17].

They are particularly important for the refugee camp community and their governing body and they will be used to inform policy. When presenting preliminary findings of this project to local stakeholders, they expressed their concerns and acknowledged the urgency of comprehensive SRH education in the camps to better prepare their youth. Follow-up meetings were planned to address these issues. Further research needs to be done on the strategy, cultural and religious barriers, timing, and contents of comprehensive SRH education initiatives in the communities on the Thailand-Myanmar border. Informal discussions with the midwives and counsellors of the MLA refugee and MKT migrant antenatal clinic also highlighted their interest in new adolescent-friendly SRH initiatives and additional training to deliver them [33–36].

## Conclusion

Adolescent pregnancy remains a major global health issue that contributes significantly to the morbidity and mortality in this age group and can create a negative cycle of adverse health, economic and social outcomes. In this study most refugee and migrant adolescents perceive their unplanned pregnancy as a challenging life event, not only for themselves, but also for their babies and families. They describe how traditional views and stigma on sexual and reproductive health issues have contributed to an important knowledge gap on contraception and life skills necessary to negotiate sexual and reproductive choices, especially for unmarried adolescents. The perceptions and views on adolescent pregnancy presented in this study can help to identify and develop effective strategies to provide comprehensive adolescent-friendly sexual and reproductive health services and education in refugee and migrant communities on the Thailand-Myanmar border. They advocate for a more tolerant and less stigmatising environment that can encourage open communication between adolescents and their families and support shared decision making in sexual relationships.

## Abbreviations

ANC: Antenatal clinic
EGA: Estimated gestational age
FGD: Focus group discussion
HIV: Human immunodeficiency virus
MKT: Maw Ker Thai migrant clinic
MLA: Mae La refugee camp clinic
NGO: Non-governmental organisations
SDG: Sustainable development goals
SMRU: Shoklo Malaria Research Unit
SRH: sexual and reproductive health
